# TNF*α*-Mediated Necroptosis Aggravates Ischemia-Reperfusion Injury in the Fatty Liver by Regulating the Inflammatory Response

**DOI:** 10.1155/2019/2301903

**Published:** 2019-05-12

**Authors:** Faji Yang, Longcheng Shang, Shuai Wang, Yang Liu, Haozhen Ren, Wei Zhu, Xiaolei Shi

**Affiliations:** ^1^Department of Hepatobiliary Surgery, Affiliated Drum Tower Hospital of Nanjing University Medical School, 321 Zhongshan Road, Nanjing, 210008 Jiangsu Province, China; ^2^Department of Anesthesiology, Affiliated Drum Tower Hospital of Nanjing University Medical School, 321 Zhongshan Road, Nanjing, 210008 Jiangsu Province, China

## Abstract

Nonalcoholic fatty liver disease (NAFLD) is more sensitive to ischemia and reperfusion injury (IRI), while there are no effective methods to alleviate IRI. Necroptosis, also known as “programmed necrosis,” incorporates features of necrosis and apoptosis. However, the role of necroptosis in IRI of the fatty liver remains largely unexplored. In the present study, we aimed to assess whether necroptosis was activated in the fatty liver and whether such activation accelerated IRI in the fatty liver. In this study, we found that the liver IRI was enhanced in HFD-fed mice with more release of TNF*α*. TNF*α* and supernatant of macrophages could induce necroptosis of hepatocytes *in vitro*. Necroptosis was activated in NAFLD, leading to more severe IRI, and such necroptosis could be inhibited by TN3-19.12, the neutralizing monoclonal antibody against TNF*α*. Pretreatment with Nec-1 and GSK′872, two inhibitors of necroptosis, significantly reduced the liver IRI and ROS production in HFD-fed mice. Moreover, the inhibition of necroptosis could decrease ROS production of hepatocytes *in vitro*. Inflammatory response was activated during IRI, and necroptosis inhibitors could suppress signaling pathways of inflammation and the soakage of inflammation cells. In conclusion, TNF*α*-induced necroptosis played an important role during IRI in the fatty liver. Our findings demonstrated that necroptosis might be a potential target to reduce the fatty liver-associated IRI.

## 1. Introduction

Hepatic ischemia-reperfusion injury (IRI) occurs under a variety of clinical conditions, such as liver transplantation and resection, as well as hemorrhagic shock [1, 2]. IRI emerges due to not only the depletion of oxygen and ATP during hypoxia but also an excessive inflammatory response after reperfusion, leading to cell death, including apoptosis, necrosis, and ultimately organ dysfunction [3]. Although the nature of hepatic IRI has been widely studied, the molecular mechanism underlying the hepatocyte death remains largely unexplored. Nonalcoholic fatty liver disease (NAFLD) is the most common cause of chronic liver disease in Western countries, and it is predicted to become also the most frequent indication for liver transplantation by 2030 [4]. Both clinical studies and animal experiments have found that the steatotic liver is particularly susceptible to IRI [5, 6]. Currently, as the main source of marginal donors, livers with greater than 30% of macrovesicular fat are considered unsuitable for transplantation due to their increased susceptibility to IRI and greater risk of early graft dysfunction [7]. The productions of proinflammatory cytokines, TNF*α* and IL1*β*, are increased during IRI, which are two critical mediators in the fatty liver [8]. TNF*α* plays a crucial role in almost all the pathogenic nodes of NAFLD, such as development of hepatic steatosis [9], hepatocyte death [10], and fibrosis [11]. However, whether proinflammatory cytokines, such as TNF*α*, are involved in regulating IRI in the fatty livers remains unknown.

Necroptosis is a novel mode of cell death, known as “programmed necrosis,” which incorporates features of necrosis and apoptosis, and such type of cell death is controlled by two kinase receptor-interacting proteins (RIP1 and RIP3) [12]. At the functional level, the auto- and transphosphorylations of RIP1 and RIP3 are required for necrosome assembly and activation of necroptotic signaling [13]. RIP3 recruits and phosphorylates the mixed lineage kinase domain-like protein (MLKL), which in turn oligomerizes and causes irreversible cellular membrane damage, resulting in necrotic cell death [14]. It has been suggested that MLKL increases the production of mitochondrial reactive oxygen species (ROS) through mitochondrial targets. Accumulating evidence indicates that necroptosis plays a crucial role in the pathogenesis of inflammatory diseases, including NAFLD [15, 16]. A study has found that necroptosis is best characterized in the setting of TNF*α*-induced cell death, which has high relevance for many types of liver diseases, but it may also occur under other conditions, including IRI [17]. It has already been shown that the inhibition of necroptosis attenuates necrotic cell death in cardiac, renal, and brain IRI as well as in the liver [18–21]. However, it remains unknown whether necroptosis can be activated by TNF*α*, and the role of necroptosis during IRI in the fatty liver is also unclear.

In the present study, we found that IRI and ROS production were more serious in the fatty liver compared with the normal liver. Macrophages stimulated with fatty acid expressed and released more TNF*α* during IRI both *in vivo* and *in vitro*. Moreover, necroptosis was activated in hepatocytes stimulated with TNF*α* or supernatant from palmitic acid- (PA-) treated macrophages followed by hypoxia-reoxygenation (H/R) injury. Necroptosis inhibitors necrostatin-1 (Nec-1) and GSK′872 could protect livers from IRI in both CD- and HFD-fed mice. In addition, Nec-1 and GSK′872 reduced the ROS level induced by IRI. Furthermore, the inhibition of necroptosis could alleviate inflammatory reaction. Collectively, we, for the first time, investigated the roles of necroptosis during IRI in the fatty liver and provided a potential target to alleviate the fatty liver-associated IRI in liver surgery.

## 2. Materials and Methods

### 2.1. Animals

Experiments were conducted using male C57BL/6J mice, which were purchased from the Animal Center of the Affiliated Drum Tower Hospital of Nanjing University Medical School and housed under specific pathogen-free conditions. The animal protocols were approved by the Institutional Animal Care and Use Committee of Nanjing University, China, based on the NIH *Guide for the Care and Use of Laboratory Animals*. All efforts were made to minimize suffering of animals.

Male C57BL/6 mice (3-4 weeks old) were fed with a high-fat diet (HFD: 60% fat, 20% protein, and 20% carbohydrates; 520 kcal/100 g; D12492; Research Diets, New Brunswick, NJ, USA) for 14 weeks to induce steatosis.

### 2.2. Mouse Hepatic IR Injury

Briefly, 70% hepatic warm ischemia of mice was induced as previously described [22]. After anesthesia, the hepatic artery, portal vein, and bile duct branches to the left and median liver lobes were clamped for 60 min. Mice were sacrificed after 6 h, and liver and serum samples were collected. Blood samples were analyzed immediately using an automatic analyzer (Fuji, Tokyo, Japan) for alanine aminotransferase (ALT) and aspartate aminotransferase (AST). The livers were cut into pieces and preserved in 4% formalin or snap frozen in liquid nitrogen.

### 2.3. Cell Culture

Kupffer cells (KCs) were isolated as previously described [22]. Briefly, livers were perfused in situ via the portal vein with CMF-HBSS, followed by 0.02% type IV collagenase in HBSS. Then, the liver was dissociated in 0.02% type IV collagenase and filtered through sterile nylon gauze to remove undigested tissue and connective tissue, followed by centrifugation at 35g for 3 min for two times to separate nonparenchymal cells (NPCs). NPCs were then suspended in HBSS and layered onto a 50/25% two-step Percoll gradient (Sigma-Aldrich, USA) in a 50 mL conical centrifuge tube and centrifuged at 1,800g for 15 min at 4°C. KCs in the middle layer were collected and allowed attaching onto cell culture plates in RPMI 1640 medium containing 1% penicillin/streptomycin and 10% fetal bovine serum (FBS).

For primary mouse hepatocytes, cell suspension was washed in William's medium supplemented with 100 nM dexamethasone, 2 mM L-glutamine, 1 *μ*M insulin, 10% FBS, and 1% penicillin/streptomycin (attachment medium) twice. The hepatocyte suspension was plated on rat tail collagen I-coated six-well plates in the attachment medium. The cells were incubated for 4 h, and then they were washed and further incubated in William's medium supplemented with 2 mM L-glutamine, 10% FBS, and 1% penicillin/streptomycin. Primary hepatocytes were used for experiments within 2-3 days after isolation.

For evaluation of H/R injury in vitro, cells were treated with PA (Sigma-Aldrich, USA) for 16 h, followed by fluxing with 95% N_2_/5% CO_2_ in the absence of FBS and incubation at 37°C for 16 h. For reoxygenation, cells were transferred to a 95% air/5% CO_2_ gas mixture and 10% FBS was added.

### 2.4. Western Blotting Analysis

Proteins were subjected by SDS/PAGE (12% or 10% gel), and the blots were incubated overnight with primary antibodies. The following primary antibodies were used: anti-RIP1 (Cell Signaling Technology, #3493), anti-RIP3 (Santa Cruz Biotechnology, sc-374639), anti-MLKL (phospho S345) (Abcam, ab196436), anti-MLKL (Cell Signaling Technology, #37705), anti-JNK1+JNK2+JNK3 (Abcam, ab208035), anti-JNK1+JNK2+JNK3 (phospho T183+T183+T221) (Abcam, ab124956), anti-c-Jun (Abcam, ab32137), anti-c-Jun (phospho S73) (Abcam, ab30620), anti-ERK1+ERK2 (Abcam, ab17942), anti-ERK1 (pT202/pY204)+ERK2 (pT185/pY187) (Abcam, ab50011), anti-p38 (Abcam, 170099), anti-p38 (phospho Y182) (Abcam, ab47363), anti-NF-*κ*B (Abcam, ab16502), anti-NF-*κ*B (phospho S536) (Abcam, ab86299), anti-IKB*α* (Abcam, ab32518), anti-IKB*α* (phospho S36) (Abcam, ab133462), and anti-GAPDH (Abcam, ab181603).

### 2.5. Quantitative Real-Time Polymerase Chain Reaction (qRT-PCR)

Hepatocyte RNA was extracted from snap-frozen liver tissues with TRIzol™ reagent (Life Technologies, USA) according to the manufacturer's instructions. Reverse transcription was performed with PrimeScript™ RT Master Mix (Takara, Japan) according to the manufacturer's instructions. qRT-PCR was performed using TB Green™ *Premix Ex Taq*™ (Takara, Japan) and ABI Prism 7500 real-time PCR System (Applied Biosystems, USA). Primers used for qPCR are as follows: *β*-actin forward: 5′-AGTGTGACGTTGACATCCGTA-3′, reverse: 5′-GCCAGAGCAGTAATCTCCTTCT-3′ and TNF*α* forward: 5′-GACGTGGAACTGGCAGAAGAG-3′, reverse: 5′-ACCGCCTGGAGTTCTGGAA-3′.

### 2.6. Enzyme-Linked Immunosorbent Assay (ELISA)

The levels of TNF*α* (eBioscience, USA) in mouse serum and cell culture supernatants were measured using commercially available ELISA kits according to the manufacturer's instructions.

### 2.7. Histological and Immunohistochemical Analysis

Paraffin liver sections (5 *μ*m) were stained with hematoxylin and eosin (HE) for histological evaluation of IRI based on standard pathology methods and visualized using a light microscope. Liver damage was evaluated using Suzuki's score by two independent pathologists. To identify macrophages and neutrophils, paraffin-embedded liver sections (5 *μ*m) were stained with F4-80 (Abcam, USA) and myeloperoxidase (MPO, Abcam, USA) as previously described [22].

### 2.8. PI Staining

Cells and frozen liver sections (4 *μ*m) were fixed with 4% paraformaldehyde for 30 min and washed twice with PBS. After treatment with 10 mg/mL DNase-free RNase at 37°C for 30 min, cell nuclei were stained with 10 mg/mL propidium iodide (PI, KeyGEN BioTECH, China) at room temperature for 5 min in the dark, then counterstained with DAPI, and observed under a fluorescence microscope.

### 2.9. Determination of ROS

For liver tissues, the ROS level was measured with the dihydroethidium (DHE, KeyGEN BioTECH, China) following the manufacturer's instructions. Briefly, frozen liver sections (4 *μ*m) were incubated with 20 *μ*M DHE in the dark at 37°C for 30 min and then counterstained with DAPI. After washing, slides were mounted and observed under an immunofluorescence microscope.

For cells, cells were incubated in the dark with 10 *μ*mol/L DCFH-DA (Beyotime Institute of Biotechnology, China) at 37°C for 20 min and then washed with PBS three times to remove residual probes. DCFH-DA was intracellularly by nonspecific esterase and oxidized by oxidant species to form the fluorescent compound 2′,7′-dichloro-fluorescein (DCF). The fluorescent signal intensity of DCF was detected under an immunofluorescence microscope.

### 2.10. Oil Red O Stain Assay

To detect lipid accumulation in macrophages, Oil Red O Stain Kit (Jiancheng Bioengineering Institute, China) was used according to the manufacturer's instructions and visualized using a light microscope.

### 2.11. Immunocytofluorescence (ICF) Analysis

Immunofluorescence analysis was performed according to the previously described protocols. Briefly, frozen liver sections (4 *μ*m) fixed with acetone were penetrated with 0.3% Triton for 15 min. Then, the slides were blocked with 10% fetal sheep serum, followed by incubation with primary antibodies overnight at 4°C. After washing, slides were incubated with the corresponding secondary antibodies, followed by incubation with DAPI. Representative images were observed under an immunofluorescence microscope. The following antibodies were used: anti-RIP3 (Santa Cruz Biotechnology, sc-374639) and goat anti-mouse IgG H&L (Alexa Fluor® 488) (Abcam, ab150117).

### 2.12. Statistical Analysis

Statistical analysis was performed using the GraphPad Prism software version 6.0. All data were expressed as mean ± standard error of the mean (SEM). Normally distributed data were tested by Student's *t*-test. *P* value less than 0.05 was considered statistically significant.

## 3. Results

### 3.1. Expression and Secretion of TNF*α* Are Increased in the Fatty Liver after IRI

**Figure 1:**
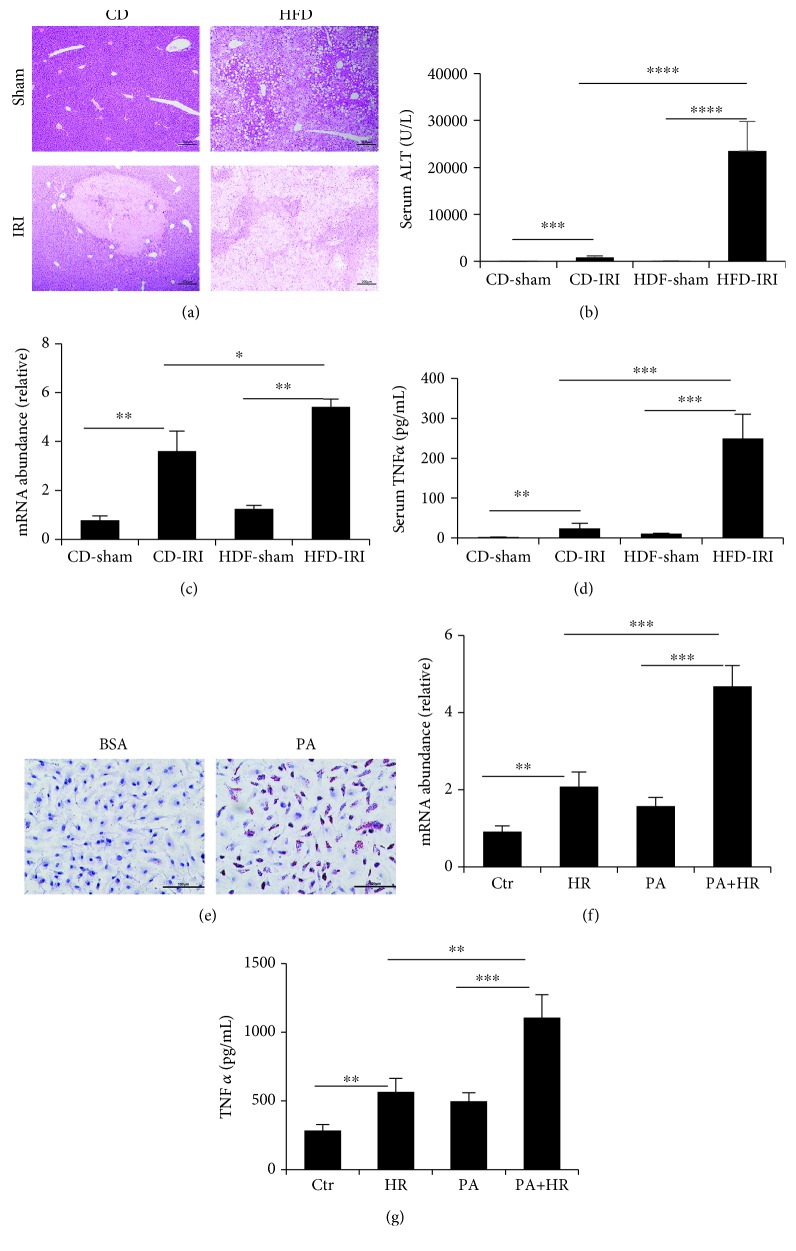
Expression and secretion of TNF*α* are increased in the fatty liver after IRI. (a) Representative H&E staining of liver sections of CD- and HFD-fed mice after IR. Scale bars, 200 *μ*m. (b) Serum ALT of CD- and HFD-fed mice after IR were measured (*n* = 6 − 8). (c) qPCR analysis of TNF*α* of CD- and HFD-fed mice after IR (*n* = 4 − 5 per group). (d) Serum TNF*α* was measured after IR in CD- and HFD-fed mice (*n* = 5 − 6 per group). (e) Representative Oil Red O staining of KCs treated with PA (500 *μ*M, Sigma-Aldrich, USA) or PBS for 24 h. Scale bars, 100 *μ*m. (f) TNF*α* in cell supernatant were measured (*n* = 6 per group). (g) qPCR analysis of TNF*α* in KCs treated with PA followed by H/R. Data are mean ± SEM; ^∗^*P* < 0.05, ^∗∗^*P* < 0.01, ^∗∗∗^*P* < 0.001, and ^∗∗∗∗^*P* < 0.0001 by unpaired Student's *t*-test.

Compared with the control group (fed with a control diet, CD), the mice fed with a HFD exhibited significantly worse IRI, evidenced by higher serum ALT and increased Suzuki's score in HFD-fed mice (Figures 1(a) and 1(b) and Table 1). From the pathological liver sections, there were more edema, sinusoidal congestion, and necrosis in HFD-fed mice (Figure 1(a)). The expression of TNF*α* at both the liver tissue and serum levels was higher in HFD-fed mice compared with those fed with a CD (Figures 1(c) and 1(d)). Macrophages are the major source of inflammatory factors, including TNF*α*. Therefore, we extracted KCs and treated with PA (50 *μ*m) to simulate macrophages in the fatty liver. After 24 h of stimulation, lipid accumulation was found in the cytoplasm (Figure 1(e)). Then, we investigated the effect of IRI on KCs with steatosis using an *in vitro* model of IRI. After H/R stimulation, the expression of TNF*α* at the mRNA level in KCs and cellular supernatant was increased. Moreover, PA treatment enhanced the expression of TNF*α* (Figures 1(f) and 1(g)). In conclusion, macrophages in the fatty liver expressed and released more TNF*α* compared with the normal liver after IRI.

### 3.2. TNF*α* Induces Necroptosis *In Vitro*

**Figure 2:**
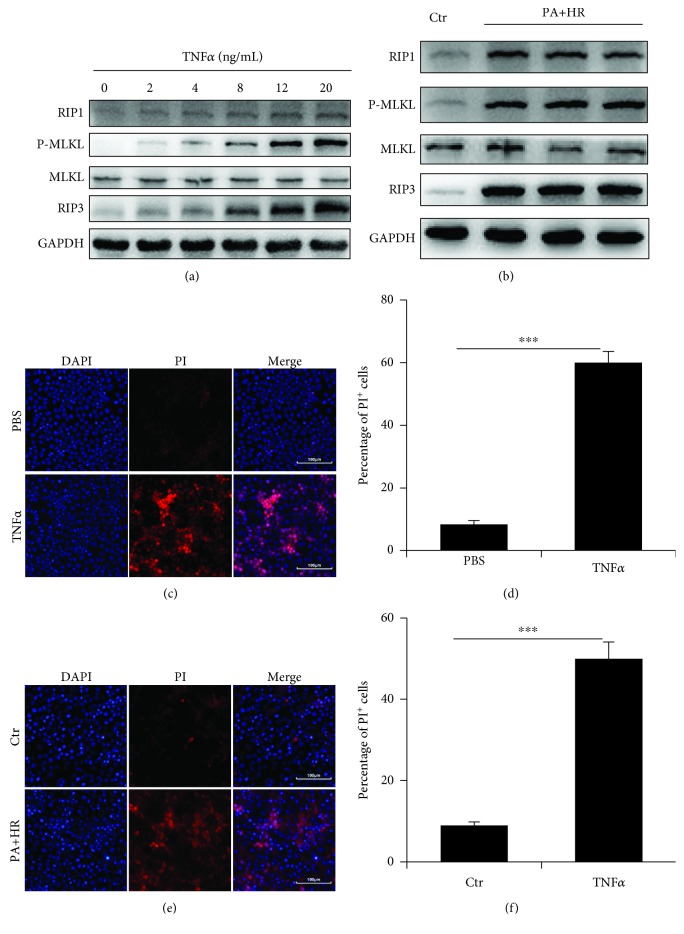
TNF*α* induces necroptosis *in vitro*. Hepatocytes were cultured with TNF*α* (PeproTech, USA) or cell supernatant of KCs treated with PA followed by H/R for 24 h. (a) Immunoblot analysis of RIP1, RIP3, and MLKL of hepatocytes treated with different concentrations of TNF*α*. (b) Immunoblot analysis of RIP1, RIP3, and MLKL of hepatocytes treated with cell supernatant of KCs for 24 h. (c, d) Representative immunofluorescence staining of propidium iodide (PI) staining of hepatocytes treated with TNF*α* (20 ng/mL) for 24 h. Scale bars, 100 *μ*m. (e, f) Representative immunofluorescence staining of PI of hepatocytes treated with cell supernatant of KCs for 24 h. Scale bars, 100 *μ*m. Data are mean ± SEM; ^∗^*P* < 0.05, ^∗∗^*P* < 0.01, and ^∗∗∗^*P* < 0.001 by unpaired Student's *t*-test.

Studies have shown that necroptosis is best characterized in the setting of TNF*α*-induced cell death. To further verify whether necroptosis was associated with TNF*α*, primary mouse hepatocytes were treated with TNF*α* for 24 h.  Figure 2(a) shows that the expressions of RIP1, RIP3, and MLKL were significantly increased upon stimulation of TNF*α* and necroptosis was induced by TNF*α* in a concentration-dependent manner. Cellular supernatant of KCs treated with PA and H/R could also activate necroptosis of hepatocytes (Figure 2(b)). The viability of hepatocytes was assessed by dual staining of DAPI and PI. A high proportion of PI^+^ cells was found after TNF*α* treatment (Figures 2(c) and 2(d)). The number of PI^+^ cells was also markedly increased when stimulated with supernatant of KCs treated with PA and H/R (Figures 2(e) and 2(f)). Therefore, these results showed that TNF*α* could induce necroptosis *in vitro*.

### 3.3. Necroptosis Is Found in NAFLD after IRI

**Figure 3:**
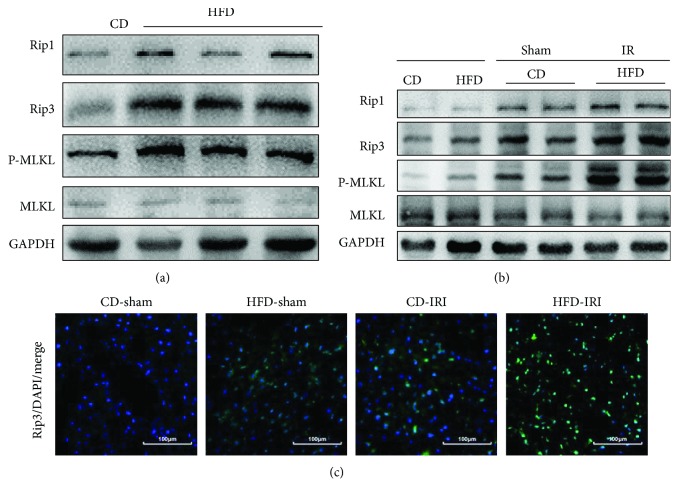
Necroptosis is found in NAFLD after IRI. (a) Immunoblot analysis of necroptosis markers RIP1, RIP3, and MLKL of mice fed with a CD or a HFD. (b) Immunoblot analysis of RIP1, RIP3, and MLKL of CD- and HFD-fed mice after 60 min of ischemia and 6 h of reperfusion. (c) Representative immunofluorescence staining of RIP3 was performed in CD- and HFD-fed mice with or without 60 min of ischemia and 6 h of reperfusion. Scale bars, 100 *μ*m.

We showed TNF*α*-induced necroptosis *in vitro*, and then we detected necroptosis in the fatty liver following IRI.  Figure 3(a) reveals that necroptosis was activated in livers of HFD-fed mice. After IRI, necroptosis was further activated, exhibiting the upregulation of necroptotic markers (RIP1, RIP3, and MLKL). This finding suggested that necroptosis was further activated by IRI (Figure 3(b)). Moreover, immunofluorescence staining of RIP3 further indicated that necroptosis was activated in NAFLD with or without IRI (Figure 3(c)). Taken together, necroptosis was activated in the fatty liver and further enhanced after IRI, which might contribute to the enhanced IRI in HFD-fed mice.

### 3.4. Inhibition of Necroptosis Reduces IRI in NAFLD

**Figure 4:**
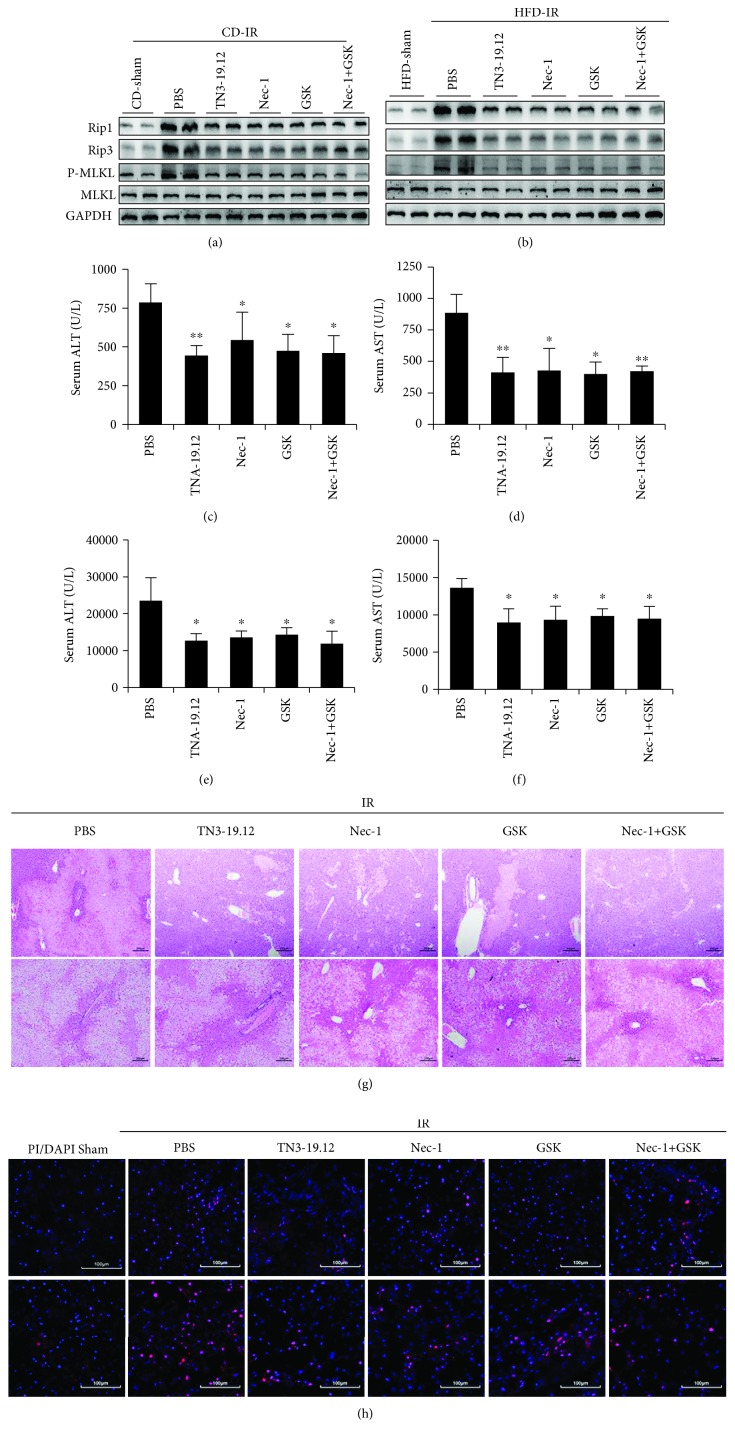
Inhibition of necroptosis reduces ischemia-reperfusion injury of NAFLD. (a) Immunoblot analysis of necroptosis markers RIP1, RIP3, and MLKL of mice fed with a CD after IRI pretreated with Nec-1 (1.65 mg/kg, Selleck, USA), GSK′872 (1.9 mM/kg, Selleck, USA), and TN3-19.12 (250 *μ*g/mouse, Sigma-Aldrich, USA). (b) Immunoblot analysis of necroptosis markers RIP1, RIP3, and MLKL of mice fed with HFD after IRI pretreated with TN3-19.12, Nec-1, and GSK′872. (c, d) Serum ALT and AST of mice fed with a CD after IRI (*n* = 6 − 8 per group). (e, f) Serum ALT and AST of mice fed with a HFD after IRI (*n* = 6 − 8 per group). (g) Representative H&E staining of liver sections. Scale bars, 200 *μ*m. (h) Representative immunofluorescence staining of PI. Scale bars, 100 *μ*m. Data are mean ± SEM; ^∗^*P* < 0.05 by unpaired Student's *t*-test.

Necroptosis was activated during IRI, and the fatty liver further aggravated the activation. Therefore, we speculated whether the inhibition of necroptosis could attenuate IRI in the fatty liver. Nec-1 and GSK′872, two necroptosis inhibitors, were administered by intraperitoneal injection 1 h before IRI. TN3-19.12, the neutralizing monoclonal antibody against TNF*α* [23], was also used to confirm whether TNF*α* was an effective trigger of necroptosis during liver IRI by intraperitoneal injection. We found that TN3-19.12, Nec-1, and GSK′872 could significantly inhibit necroptosis in both CD- and HFD-fed mice, showing increased expressions of RIP1, RIP3, and MLKL (Figures 4(a) and 4(b)). Consistent with our conjecture, Nec-1 and GSK′872 as well as TN3-19.12 reduced levels of ALT and AST in CD- and HFD-fed mice (Figures 4(c)–4(f)). HE staining and decreased Suzuki's scores revealed that liver injury was also reduced (Figure 4(g) and Tables 2 and 3). PI staining exhibited that hepatocyte necrosis, the direct result of IRI damage, was also alleviated after IRI (Figure 4(h)).

**Figure 5:**
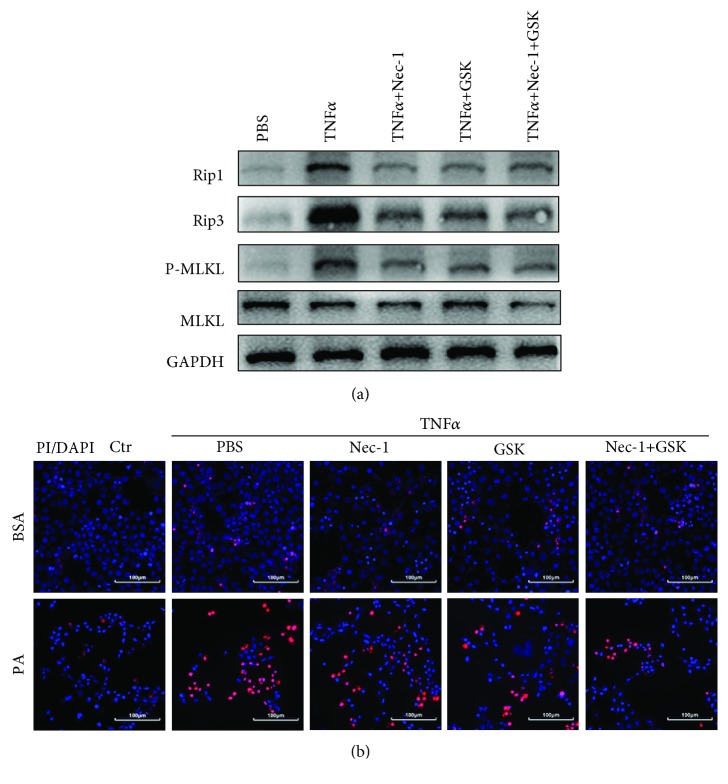
Inhibition of necroptosis reduces hepatocyte death induced by TNF*α in vitro*. (a) Hepatocytes were cultured with TNF*α* (20 ng/mL) for 24 h. Immunoblot analysis of necroptosis markers RIP1, RIP3, and MLKL of hepatocytes pretreated with Nec-1 (100 *μ*M) and GSK′872 (5 *μ*M). (b) Representative immunofluorescence staining of PI. Scale bars, 100 *μ*m.

We also studied the above-mentioned conjecture *in vitro*. Primary hepatocytes were stimulated with TNF*α* to induce IRI. Nec-1 and GSK′872 could also reduce the expressions of RIP1, RIP3, and MLKL in TNF*α*-treated hepatocytes (Figure 5(a)). Furthermore, Nec-1 and GSK′872 also decreased the proportion of PI^+^ hepatocytes (Figure 5(b)). Taken together, the inhibition of necroptosis could reduce cell injury induced by TNF*α* during IRI in NAFLD.

### 3.5. Inhibition of Necroptosis Reduces ROS Production after IRI in NAFLD Both *In Vivo* and *In Vitro*

**Figure 6:**
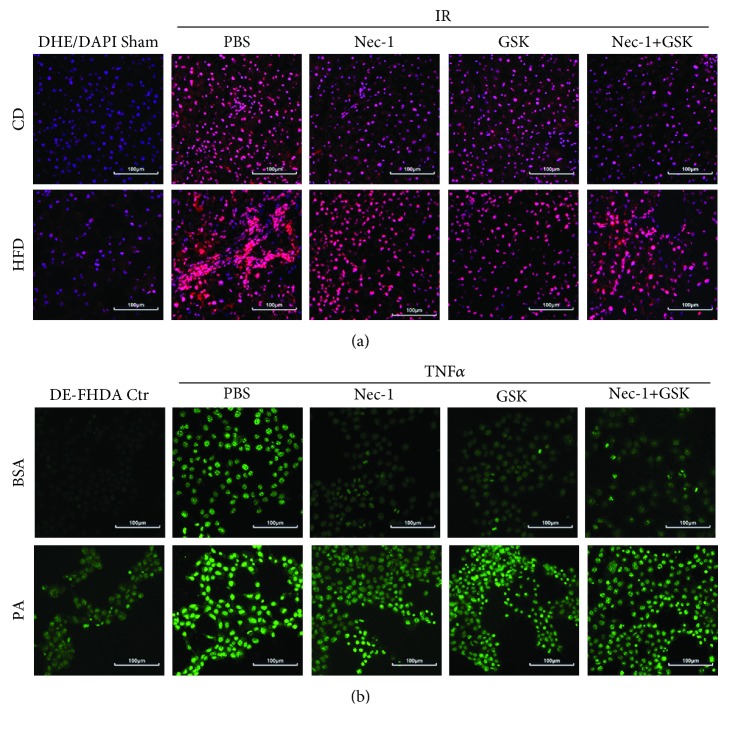
Inhibition of necroptosis reduces ROS after IRI of NAFLD both *in vivo* and *in vitro*. (a) Representative images of DHE staining of liver sections in CD- and HFD-fed mice with Nec-1 and GSK′872 pretreatment. Scale bars, 100 *μ*m. (b) Representative images of DE-FHDA staining of hepatocytes with Nec-1 and GSK′872 pretreatment followed by TNF*α* stimulation. Scale bars, 100 *μ*m.

The pathophysiology of hepatic IRI generally includes ROS production. In terms of entity of oxidative stress, the most relevant event is ROS production by activated inflammatory cells, while liver cells can also produce ROS by the uncoupled mitochondria due to oxygen readmission [24]. Moreover, it has been suggested that MLKL increases mitochondrial ROS production through mitochondrial targets [25]. Therefore, we detected whether Nec-1 and GSK′872 could reduce the ROS production.  Figure 6(a) reveals that ROS production was increased during IRI, and it was further enhanced in the fatty liver. Pretreatment with Nec-1 and GSK′872 could significantly decrease the ROS level. The same results were also found by *in vitro* experiments (Figure 6(b)). Therefore, the inhibition of necroptosis could reduce the ROS level after IRI in NAFLD, and this might be another mechanism of alleviating IRI in the fatty liver.

### 3.6. Inhibition of Necroptosis Reduces the Inflammatory Response after IRI in NAFLD

**Figure 7:**
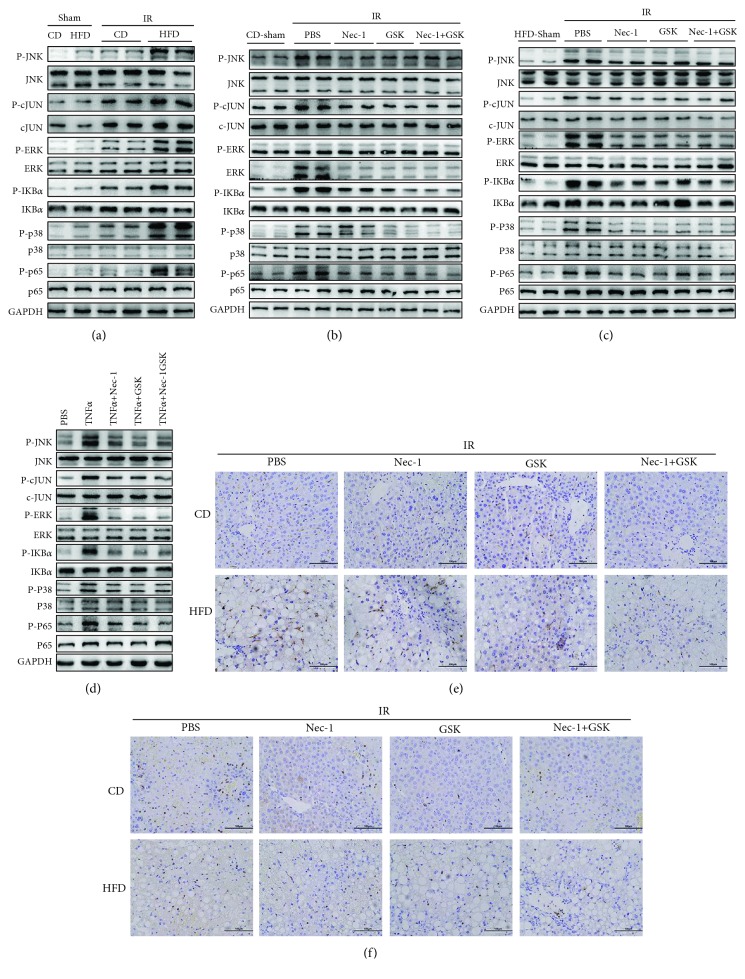
Inhibition of necroptosis reduces the inflammatory response after IRI of NAFLD. (a) Immunoblot analysis of JNK, cJUN, ERK, IKB*α*, p38, and p65 of CD- and HFD-fed mice with or without IRI. (b) Immunoblot analysis of JNK, cJUN, ERK, IKB*α*, p38, and p65 of CD-fed mice after IRI with Nec-1 and GSK′872 pretreatment. (c) Immunoblot analysis of JNK, cJUN, ERK, IKB*α*, p38, and p65 of CD-fed mice after IRI with Nec-1 and GSK′872 pretreatment. (d) Immunoblot analysis of JNK, c-JUN, ERK, IKB*α*, p38, and p65 of hepatocytes with Nec-1 and GSK′872 pretreatment. (e) Representative F4-80 immunohistochemistry of liver sections with IRI in CD- and HFD-fed mice. Scale bars, 100 *μ*m. (f) Representative MPO immunohistochemistry of liver sections with IRI in CD- and HFD-fed mice. Scale bars, 100 *μ*m.

Danger-associated molecular patterns (DAMPs) are either passively released by necrotic cells and the damaged extracellular matrix or are actively secreted by stressed and injured cells [26]. Various types of DAMPs are released during liver IRI, and these DAMPs can interact with and activate toll-like receptor (TLRs). The TLRs are one of the components by which the innate immune system senses the invasion of pathogenic microorganisms or tissue damage by recognizing DAMPs [27]. Transcription factors have been shown to mediate TLR activation in liver IRI, including NF-*κ*B, JNK, ERK, p38, and IKB*α* [28]. Therefore, we assessed the expressions of these transcription factors in the fatty livers.  Figure 7(a) shows that compared with CD-fed mice, all the transcription factors were activated in HFD-fed mice and IRI increased the expressions of NF-*κ*B, JNK, ERK, p38, and IKB*α*. Moreover, we found that the liver inflammatory response after IRI was inhibited by Nec-1 and GSK′872 in both CD- and HFD-fed mice (Figures 7(b) and 7(c)). In addition, the same findings were achieved by *in vitro* experiments (Figure 7(d)). There was also more infiltration of MPO- and F4-80-positive cells in HFD-fed mice after IRI. Inhibition of necroptosis could also decrease the soakage of inflammation cells (Figures 7(e) and 7(f)). In summary, the inhibition of necroptosis by Nec-1 and GSK′872 could reduce the inflammatory response after IRI in NAFLD, which might be another mechanism protecting the fatty liver from IRI.

## 4. Discussion

IRI results from a prolonged ischemic insult, followed by the restoration of blood perfusion. Hepatic IRI can lead to severe liver injury, and it is a major cause for the failure of liver transplantation. However, the fatty liver is more sensitive to IRI, leading to more severe outcomes of patients. Moreover, in the past two decades, urbanization has led to sedentary lifestyle and overnutrition, setting the stage for the epidemic of obesity and NAFLD, which is currently estimated to be 24% worldwide [29, 30]. Therefore, it is urgently necessary to prevent and attenuate IRI [22, 31]. However, there are no available effective and simple methods available to reduce IRI in the fatty liver. In the present study, we also found that IRI in the fatty liver was more severe compared with the normal liver and the TNF*α* level was increased in serum and liver of NASH animals, which was in agreement with the previous report [11].

**Figure 8:**
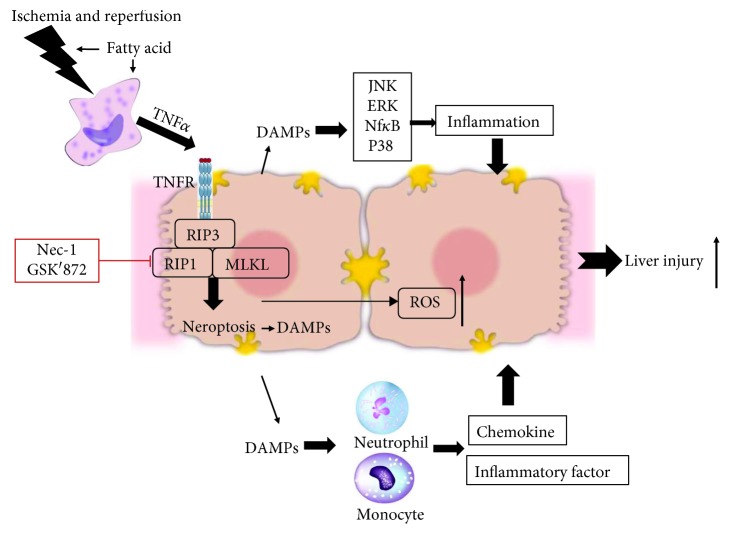
TNF*α*-mediated necroptosis aggravates IRI in the fatty liver by regulating the inflammatory response. Macrophages were activated during IRI, which secreted more TNF*α* in the fatty liver compared with the normal liver. Necroptosis induced by TNF*α* was activated in NAFLD, leading to more severe IRI. Necroptosis promoted the production of DAMPs, which increased signaling pathways of inflammation and the soakage of inflammation cells to aggravate IRI. Inhibition of necroptosis with Nec-1 and GSK′872 could decrease the signaling pathway of inflammation and ROS production to protect the liver from IRI.

As a newly defined type of programmed cell death, necroptosis is tightly controlled by the multiprotein complex of RIP1 and RIP3, known as the necrosome. Accumulating evidence indicates that MLKL and the protein kinases (RIPK1 and RIPK3) contribute to inflammatory processes through both the induction of necroptotic cell death and other cellular changes [32, 33]. Necroptosis has been shown to be involved in various ischemic, inflammatory, and neurodegenerative human disorders [34]. Necroptosis has been identified as a mechanism of cell death in renal, cardiac, and retinal IRI [18, 35, 36]. A recent study has found that necroptosis contributes to hepatic damage during IR, which induces autophagy via ERK activation [21]. However, another study has found that necroptotic molecules are not increased in the necrosis-dominant hepatic IRI model, and antinecroptosis does not have an overall protective effect on necrosis-dominant hepatic IRI [37]. Therefore, the role of necroptosis or even whether necroptosis is activated in liver IRI remains largely unexplored. In the present study, we found that TNF*α* was upregulated in the fatty liver and its level was further increased after IRI. Early studies have found that TNF*α* is the best characterized activator to induce necroptosis. Therefore, we stimulated hepatocytes with TNF*α*. As expected, necroptosis was significantly activated by TNF*α* as well as supernatant of KCs treated with PA and H/R. Moreover, we tested the expressions of RIP1, RIP3, and MLKL in liver tissues suffering IRI, and all three markers of necroptosis were upregulated. In addition, the activation of necroptosis was much more intensive in the fatty liver, which was consistent with the level of TNF*α*. Furthermore, Nec-1 and GSK′872 could significantly reduce necroptosis and protect the liver from IRI in both CD- and HFD-fed mice (Figure 8). To the best of our knowledge, we, for the first time, demonstrated that necroptosis was activated during IRI in the fatty liver, and inhibition of necroptosis could reduce IRI in NAFLD.

Immune cells, such as macrophages, are activated during the ischemic phase and even more during reperfusion. Once activated, they produce proinflammatory cytokines, including TNF*α* [38]. Cytokines play critical roles by stimulating hepatocytes to produce ROS, greatly contributing to their damage [39]. In the present study, we also found that ROS production was increased in hepatocytes of the fatty liver after IRI as well as hepatocytes stimulated with TNF*α*. Inhibition of necroptosis could reduce the level of ROS. Therefore, necroptosis contributed to the ROS production, which might aggravate the IRI in the fatty liver. DAMPs released during liver IRI bind to a group of receptors termed pattern recognition receptors (PRRs) to induce the inflammatory response [40]. Transcription factors, including NF-*κ*B, JNK, ERK, p38, and IKB*α*, participate in the activation of the inflammatory response. In this study, we found that transcription factors, as well as the soakage of inflammation cells, were all significantly upregulated during IRI in the fatty liver, while the inhibition of necroptosis could reduce the inflammatory response (Figure 8).

## 5. Conclusions

In the present study, we found a new mechanism, which could explain why the fatty liver was more susceptible to IRI, and demonstrated the mechanism underlying the necroptosis of the fatty liver. Our findings provided a potential target to reduce the fatty liver-associated IRI in liver transplantation.

## Figures and Tables

**Table 1:** The mice fed with a HFD exhibited significantly increased Suzuki's score of IRI in HFD-fed mice.

Group	Suzuki's score
CD-sham	0.46 ± 0.23
CD-IRI	4.01 ± 0.26^a^
HFD-sham	2.97 ± 0.45^b^
HFD-IRI	7.13 ± 0.83^c,d^

The results are presented as the mean ± SEM of 6 to 8 animals per group. ^a^Significant difference (*P* < 0.001) versus the CD-sham group. ^b^Significant difference (*P* < 0.01) versus the CD-sham group. ^c^Significant difference (*P* < 0.01) versus the CD-IRI group. ^d^Significant difference (*P* < 0.01) versus the HFD-sham group.

**Table 2:** Inhibition of necroptosis reduces Suzuki's score of IRI in CD-fed mice.

Group	Suzuki's score
PBS-IRI	3.98 ± 0.26
TN3-19.12-IRI	1.51 ± 0.36^a^
Nec-1-IRI	1.77 ± 0.21^a^
GSK-IRI	2.11 ± 0.29^b^
Nec-1+GSK-IRI	1.62 ± 0.29^a^

The results are presented as the mean ± SEM of 6 to 8 animals per group. ^a^Significant difference (*P* < 0.01) versus the PBS-IRI group. ^b^Significant difference (*P* < 0.05) versus the PBS-IRI group.

**Table 3:** Inhibition of necroptosis reduces Suzuki's score of IRI in HFD-fed mice.

Group	Suzuki's score
PBS-IRI	7.03 ± 0.83
TN3-19.12-IRI	4.51 ± 0.57^a^
Nec-1-IRI	5.57 ± 0.48^b^
GSK-IRI	4.77 ± 0.56^a^
Nec-1+GSK-IRI	4.73 ± 0.79^b^

The results are presented as the mean ± SEM of 6 to 8 animals per group. ^a^Significant difference (*P* < 0.01) versus the PBS-IRI group. ^b^Significant difference (*P* < 0.05) versus the PBS-IRI group.

## Data Availability

The data used to support the findings of this study are available from the corresponding authors upon request.

## Abbreviations

NAFLD: Nonalcoholic fatty liver disease
IRI: Ischemia and reperfusion injury
ALT: Alanine aminotransferase
AST: Aspartate aminotransferase
KCs: Kupffer cells
TNF*α*: Tumor necrosis factor *α*
HFD: High-fat diet
CD: Control diet
PA: Palmitic acid
ROS: Reactive oxygen species
RIP1: Receptor-interacting protein 1
RIP3: Receptor-interacting protein 3
MLKL: Mixed lineage kinase domain-like protein
JNK: c-Jun NH2-terminal kinase
p38: Mitogen-activated protein kinase 14
NF*κ*B: Nuclear factor kappa B
ERK: Extracellular-regulated MAP kinase
IKB*α*: NF*κ*B inhibitor alpha
DAMPs: Danger-associated molecular patterns
Nec-1: Necrostatin-1
PRRs: Pattern recognition receptors.
